# Baculoviruses as Microbial Pesticides: Potential, Challenges, and Market Overview

**DOI:** 10.3390/v17070917

**Published:** 2025-06-27

**Authors:** Maider Martínez-Balerdi, Javier Caballero, Eduardo Aguirre, Primitivo Caballero, Inés Beperet

**Affiliations:** 1 Research and Development Department, Bioinsectis SL, Plaza Cein 5, Nave A14, 31110 Noáin, Spain; maider.martinez@bioinsectis.com (M.M.-B.); eduardo.aguirre@bioinsectis.com (E.A.); 2 Institute for Multidisciplinary Research in Applied Biology-IMAB, Universidad Pública de Navarra, 31006 Pamplona, Spain; pcm92@unavarra.es

**Keywords:** integrated pest management (IPM), baculoviruses, biological control, microbial pesticides, formulation, regulatory frameworks, sustainable agriculture, baculovirus market

## Abstract

Baculoviruses represent a promising group of microbial insecticides for the biological control of agricultural pests, particularly those within the order Lepidoptera. Their high host specificity and environmental safety make them ideal candidates for inclusion in integrated pest management (IPM) programs. This review presents a comprehensive overview of baculovirus biology, highlighting their infection mechanisms, selectivity, and ecological compatibility. Special attention is given to advances in mass production systems—both in vivo and in vitro—and formulation technologies that improve field efficacy and environmental persistence, including UV protectants and microencapsulation. Regulatory aspects are also discussed, comparing international approval pathways and highlighting the disparity between regions with supportive policies (e.g., Latin America, Asia) and those with more restrictive frameworks (e.g., the European Union). Additionally, the current global market landscape for baculovirus-based products is examined, with emphasis on recent growth, commercialized formulations, and challenges such as host resistance and the limited spectrum of action. By synthesizing findings from the scientific literature and industry reports, this review underscores the role of baculoviruses as effective, sustainable alternatives or complements to chemical insecticides in modern agriculture, contributing to the reduction in pesticide residues and environmental impact.

## 1. Introduction and Objectives

The increase in the world population and the subsequent increase in the demand for food place emphasis on food security. Plants at all stages of growth are susceptible to pest attacks, which cause significant damage and loss. Global crop losses remain a matter of concern since crop lands are damaged by more than 10,000 insect species worldwide [1]. To minimize this damage, the use of chemical insecticides has been globalized for many years, which has resulted in 200,000 people being killed worldwide, according to the World Health Organization (WHO), as a direct result of pesticide poisoning. These pesticides have been linked to adverse effects such as carcinogenicity, teratogenicity, high and acute residual toxicity, the potential to induce hormonal imbalance, and dermatological, respiratory, and reproductive problems, among other concerns [2,3,4,5,6,7,8,9,10,11]. In addition to direct damage, there are also many intersecting issues such as antimicrobial resistance, food security, climate change, and fragile health infrastructures that need to be addressed from a multisectoral and multidisciplinary perspective [12]. In the early 2000s, the WHO introduced the One Health (OH) concept, an integrated, transdisciplinary, and unifying approach aimed at sustainably balancing and optimizing the health of people, animals, and ecosystems. This concept has been gaining popularity ever since. OH addresses critical areas such as the prevention of zoonotic diseases in animals and people, the improvement in food safety and security, the reduction in antimicrobial-resistant infections, the protection of global health security, and the preservation of biodiversity and conservation efforts [13]. To further enhance its comprehensive approach, OH philosophy aligns with the principles of integrated pest management (IPM), promoting a holistic and interconnected strategy for health and environmental sustainability [14].

The high fertility, short life cycle, and diapause capacity of polyphagous and migratory insect pests compromise the viability of agricultural crops. Specifically, invasive insects (those encroaching upon territories where they were not previously present) are responsible for significant losses, estimated at least USD 70 billion annually. This estimate represents only a portion of the total economic impact, as the losses attributed to insects in general in agriculture are even greater [15]. Insect damage is estimated to produce a crop loss of 13.6% globally [1].

**Figure 1:**
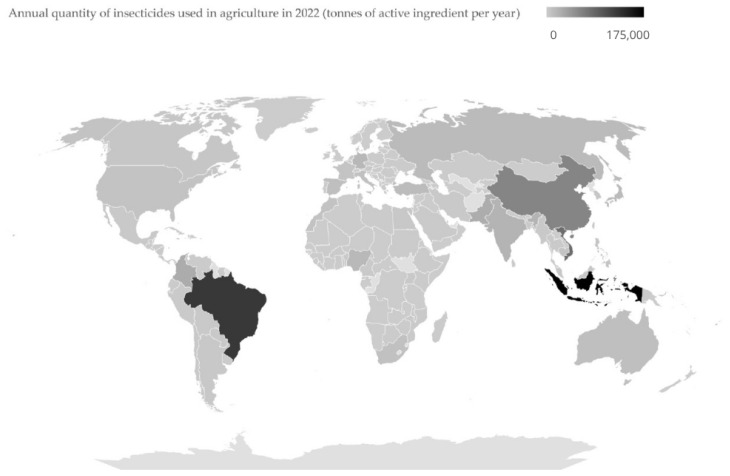
Annual quantity of insecticides used in agriculture, measured as tonnes of active ingredients per year worldwide in 2022. Data extracted from https://ourworldindata.org/grapher/insecticide-use (17 December 2024).

Controlling pests through solutions that protect the environment remains a global challenge. IPM is a strategic approach designed to mitigate the problems associated with chemical insecticides and represents a sustainable, science-driven approach to pest control that integrates biological, cultural, physical, and chemical tools. It aims to identify, manage, and mitigate risks associated with pests and pest management strategies while minimizing economic, health, and environmental impacts. This process emphasizes reducing or eliminating reliance on chemical pesticides by adopting a variety of methods that prioritize environmental and human health [16]. In 2022, the annual use of insecticides in agriculture exceeded 40,000 tonnes in the USA, Brazil, Germany, India, and China (Figure 1) [17]. Implementing IPM practices can potentially reduce this heavy dependence on chemical insecticides, thereby contributing to sustainable agriculture and environmental conservation. Biopesticides are a pivotal part of this transition and are classified into several types, including microbial pesticides, biochemical pesticides, botanical pesticides, and plant-incorporated protectants (PIPs), each with unique modes of action and applications [18].

Microbial pesticides include bacteria, viruses, microalgae, fungal, microsporidia, and apicomplexa as active ingredients, harnessing the biological processes of these microorganisms to selectively target pest species [19]. *Bacillus thuringiensis* (Bt) is a well-known example of a Gram-positive, aerobic, endospore-forming bacterium in Morphological Group I, alongside *B. cereus*, *B. anthracis*, *B. laterosporus*, and *B. subtilis* [20]. Recognized for its parasporal body (or crystal) visible within the sporangium, Bt produces toxins lethal to specific insect larvae, making it widely used in agriculture due to its specificity and safety for non-target species [21,22]. Biochemical pesticides are naturally derived substances that control pests through non-toxic mechanisms, in contrast to synthetic chemical pesticides that kill pests directly. They include insect pheromones, plant-based extracts and essential oils, and insect growth regulators (IGRs) [18]. Insect pheromones mimic natural insect chemicals to disrupt mating and reduce pest populations without killing insects [23]. Plant extracts and essential oils, such as neem and lemongrass oil, offer diverse actions like repellence, oviposition inhibition, and anti-feed effects, but require careful dosing to avoid harming non-target organisms [24,25,26,27]. IGRs, which interfere with insect development processes like molting and reproduction, are selective and less toxic to non-target species, though they are often combined with other insecticides for broader efficacy. These methods offer viable sustainable alternatives to synthetic pesticides, thereby advancing pest management strategies that are more environmentally responsible [28,29]. Plant-incorporated protectants (PIPs) are pesticidal substances produced by genetically modified organisms (GMOs), where genetic material is integrated into plants to enable them to produce these compounds. First-generation PIPs, such as Cry proteins derived from the soil bacterium Bt, were introduced into GM crops to provide insect resistance [30,31].

Among microbial biopesticides, baculoviruses have emerged as particularly valuable agents for their unique specificity and efficacy in pest management. Baculoviruses, classified under the family *Baculoviridae*, are double-stranded DNA viruses that primarily infect insects in the order Lepidoptera, including major agricultural pests such as the fall armyworm (*Spodoptera frugiperda*) [32,33,34]. The baculovirus infection process is initiated when insect larvae ingest occlusion bodies (OBs) containing the virus; these OBs dissolve in the larval midgut, releasing the virus to infect host cells and propagate throughout the larva, ultimately causing its death [35].

Baculoviruses have demonstrated significant success as biopesticides in various regions, offering environmentally friendly and species-specific pest control. Notable examples include the use of the *Anticarsia gemmatalis* multiple nucleopolyhedrovirus (AgMNPV, *Alphabaculovirus angemmatalis*) for the control of *Anticarsia gemmatalis* in soybean crops in Brazil or the use of the *Cydia pomonella* granulovirus (CpGV, *Betabaculovirus cypomonellae*) for the control of *Cydiapomonella* in apple and pear orchards worldwide [36,37]. However, although the specificity of baculoviruses offers a distinct ecological advantage, baculoviruses face challenges in adoption compared to chemical pesticides, particularly due to low speed of killing, extremely restricted host range, the need for cost-effective mass production, or stability concerns [38,39,40].

This review examines the factors involved in selecting effective baculovirus strains, including dose rates, host range, and speed of action, as well as the development of formulations to enhance product stability and efficiency. Additionally, a market overview will discuss current trends, industry leaders, and the expanding role of baculovirus-based biopesticides as environmentally friendly pest management solutions gain traction. Finally, it will address the regulatory frameworks governing biopesticides in major markets like the European Union, the United States, Brazil, and China, which impact the pathways for baculovirus commercialization.

This comprehensive approach aims to provide an understanding of the potential of baculoviruses to contribute to sustainable pest control practices, addressing practical aspects of their development, regulatory considerations, and market potential in global agriculture.

## 2. Baculoviruses as Microbial Pesticides

Baculoviruses, as microbial pesticides, offer a selective and environmentally friendly alternative to chemical pesticides, specifically targeting insect pests mainly within the order Lepidoptera. For successful field application, several criteria must be considered in the selection of effective baculovirus strains, each impacting efficacy, field stability, and practical utility.

### 2.1. Baculovirus General Characteristics

Understanding the biological intricacies of baculoviruses is crucial to fully appreciate their application and potential in pest management. Although the association of baculoviruses with the silk industry in China dates back over 5000 years, it was not until the late 1940s that rod-shaped virions were convincing, demonstrated by electron microscopy [35,41,42]. 

The *Baculoviridae* family was recently reclassified within the newly established class *Naldaviricetes* and assigned to the order *Lefavirales* [43]. Since 1971, the International Committee on Taxonomy of Viruses (ICTV) has issued ten reports providing progressive updates on viral taxonomy. In the 6th ICTV report, *Baculoviridae* was divided into two genera, Nucleopolyhedrovirus (NPV) and Granulovirus (GV), based on OB morphology [44]. Nucleopolyhedroviruses are characterized by polyhedral OBs, with diameters ranging from 0.15 to 15 µm, primarily composed of the protein polyhedrin. NPVs’ OBs enclose multiple occlusion-derived virions (ODVs), each potentially containing either a single nucleocapsid (single nucleopolyhedroviruses, SNPVs) or multiple nucleocapsids (multiple nucleopolyhedroviruses, MNPVs). In contrast, GVs typically present a singular ODV within each OB, containing a single nucleocapsid. The OBs, predominantly composed of granulin protein, have dimensions of approximately 0.13–0.5 µm [35]. Each nucleocapsid contains a single copy of the viral genome.

The sequencing of baculoviruses isolated from mosquitoes and sawflies revealed distinct genetic lineages, prompting a revision of *Baculoviridae* taxonomy [45]. Jehle et al. [46] proposed a revised taxonomic framework and nomenclature within *Baculoviridae*. Subsequently, in the 9th ICTV report, the family *Baculoviridae* was divided into four genera: *Alphabaculovirus* (specific to lepidopteran NPVs), *Betabaculovirus* (specific to lepidopteran GVs), *Deltabaculovirus* (specific to dipteran NPVs), and *Gammabaculovirus* (specific to hymenopteran NPVs) [47]. This classification scheme reflects phylogenetic relationships and differentiates genera based on host specificity, the morphological characteristics of OBs, and genetic lineage. However, over 90% of the baculovirus species described to date are specific to Lepidoptera [48]. Most commercial baculovirus-based products are targeted against pests of the order Lepidoptera, meaning they contain alphabaculoviruses or betabaculoviruses as the active ingredient. Although less common in commercial applications, products such as Leconte Virus and Abietiv, based on gammabaculoviruses, are marketed by Andermatt Canada for the control of sawflies (Hymenoptera).

**Figure 2:**
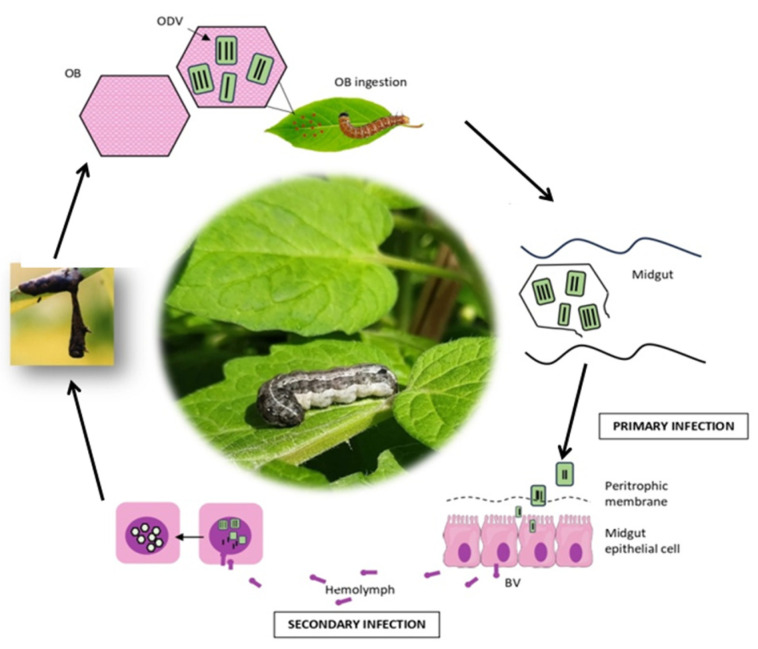
Infection cycle of baculoviruses: primary infection and secondary infection. Pink hexagons represent OBs, which contain ODVs; Green squares with black lines represent ODVs; Pink epithelial cells with surface projections and a purple nucleus represent midgut epithelial cells; Pink square cells with a central purple nucleus represent standard body cells of the insect host; Purple indicates cell nuclei throughout the figure; Green is used to depict the host insect and plant material.

Baculoviruses are typically named after the initial host from which they were isolated. For example, the *Autographa californica* multiple nucleopolyhedrovirus (AcMNPV, *Alphabaculovirus aucalifornicae*) is named after its host *Autographa californica*, the alfalfa looper [35]. AcMNPV is the most well-studied baculovirus, and its infection pathway of *Trichoplusia ni* larvae was elucidated by Granados & Lawler [49]. This study helped clarify the infection of these viruses. Baculoviruses present two distinct phenotypic forms: ODVs and budded viruses (BVs) [32,33,34,35]. In the first step of the infection cycle, after the ingestion of OBs present on contaminated crops by a susceptible host, OBs are dissolved in the midgut due to alkaline pH conditions, releasing ODVs (Figure 2). The ODVs then enter the columnar cells of the midgut epithelium, and virus replication occurs in the nucleus, completing the primary infection that leads to the production of BVs [34,50]. During the secondary infection, BVs circulate through the hemolymph and spread to other larval tissues. In the late phase of infection, the production of infectious BVs decreases, and new nucleocapsids formed in the cell nuclei acquire the ODV envelope. ODVs are subsequently occluded in a protein matrix to form new OBs [32,34,51].

Having highlighted the remarkable characteristics and infection mechanisms of baculoviruses, we can now delve into their potential as biological pesticides, addressing their limitations with innovative solutions.

### 2.2. Baculovirus Pesticides: Strengths and Weaknesses

The ecological advantages of baculoviruses are significant. Their narrow host range allows for targeted pest control, preserving biodiversity and preventing disruptions in agroecosystems [52]. Most baculoviruses are highly species-specific pathogens, such as the *Spodoptera exigua* multiple nucleopolyhedrovirus (SeMNPV, *Alphabaculovirus spexiguae*), which infects only *Spodoptera exigua* larvae. However, others can productively infect a wider range of host species, such as AcMNPV, which is able to infect over thirty insect species of Lepidoptera, including members of the genera *Heliothis*, *Trichoplusia*, and *Spodoptera*. This specificity enables the control of targeted lepidopteran hosts without harming other beneficial species [53].

The exceptional biosecurity of baculovirus-based pesticides is one of their defining features. Their high specificity and favorable safety profile make them especially well suited for IPM strategies. Unlike chemical pesticides, baculoviruses infect only targeted pest insects, thereby minimizing adverse effects on non-target organisms such as beneficial insects, humans, and other wildlife [52]. This specificity results from the infection pathway of baculoviruses, which generally restricts their impact to a narrow range of species. Target species vary in their susceptibility to infection, and the genetic basis for the host range of each baculovirus remains unclear [54,55]. This feature of baculoviruses aligns closely with the principles of the One Health concept [56,57].

Another notable strength of baculovirus-based bioinsecticides is their ability to persist in the environment after field application, mainly in the soil, forming a natural reservoir that can provide ongoing pest control without the adverse environmental impacts commonly associated with chemical pesticides [58,59]. In the final stage of the infection cycle, the larvae killed after the application of the bioinsecticide release millions of OBs into the environment [60,61]. Therefore, the soil OB reservoir relies on regular inputs from insects that succumb to the virus, the movement of OBs via precipitation as they are washed off contaminated foliage, and the decay of leaves and crop residues that carry these viral particles. A single application of the virus can lead to years of replication and epizootics [62]. This persistence allows baculoviruses to remain viable and ready to infect host insect populations over extended periods, potentially reducing the need for repeated applications and enhancing the sustainability of pest management programs [63]. In addition to soil-to-plant transfer, other mechanisms such as the reactivation of covert infections in dense insect populations [63] and the dissemination of virus particles by predators moving across the crop field [64] may also play a significant role in enhancing viral persistence and biocontrol efficacy.

Despite their stability under certain environmental conditions, baculoviruses are inherently biodegradable agents. Their viral particles, primarily composed of proteins and nucleic acids, gradually degrade when exposed to environmental factors such as ultraviolet (UV) radiation and fluctuating temperatures [58,59,65]. Unlike synthetic chemical insecticides, which may persist as residues and pose long-term risks to non-target organisms and ecosystems, baculoviruses break down naturally. Their natural biodegradability and environmental compatibility affirm their significance as secure, sustainable components in IPM strategies.

After discussing the significant advantages of baculoviruses as insecticides, it is also important to address their weaknesses. The restricted host range also limits the practical applicability of individual baculovirus strains across multiple pest species, which may require the development of multiple formulations for different pests in diverse crop systems [54,66,67]. The management of pest complexes, which consist of multiple species coexisting within the same cropping systems, presents a significant challenge for sustainable agriculture. For example, in maize production, prominent pests include *S. frugiperda* (fall armyworm), *Helicoverpa armigera* (cotton bollworm), *H. zea* (corn earworm), and *Mythimna unipuncta* (true armyworm) [68,69,70,71,72,73,74]. Similarly, in tomato cultivation, the pest complex often comprises *Tuta absoluta* (tomato leafminer), *H. armigera*, and *S. exigua* (beet armyworm) [75,76,77,78].

To date, it has not been possible to control these species with a single baculovirus formulation due to the fact that they exhibit considerable variability in their susceptibility to single baculovirus species, primarily due to differences in virus–host specificity and the biological characteristics of each species [34]. While species-specific baculoviruses offer high efficacy against individual targets, their use often requires multiple applications of different products, since growers might need a single solution to address each different pest, leading to increased economic costs and logistical complexity [79].

Broad-spectrum baculoviruses, which can target multiple species within a pest complex, provide a promising alternative. However, the effectiveness of such viruses is limited by the uneven susceptibility of different pest species within the spectrum [34]. To maximize the utility of broad-spectrum baculoviruses, optimization efforts should focus on improving dose–response efficiency across all target species, developing advanced formulations that enhance viral stability and infectivity under varying environmental conditions, and tailoring application protocols to field-specific dynamics [80,81,82,83]. Additionally, it is critical to investigate potential synergistic or antagonistic interactions among baculoviruses targeted against the different species within the pest complex, as these may influence the overall efficacy of the viral product, as demonstrated by the study of Ferrelli and Salvador [84]. Advances in bioengineering, such as the genetic modification of baculoviruses to expand their host range without compromising specificity or environmental safety, could further enhance their applicability. For example, Wu et al. (2023) successfully engineered a recombinant baculovirus (vAcRev) with an expanded host range, demonstrating the potential of genetic recombination to enhance baculovirus efficacy in non-permissive hosts [85]. However, baculovirus-based products including genetic modifications are not commercially available due to the legal restrictions existing on GMOs.

Another challenge is their low speed of kill, as baculoviruses are generally slower-acting than synthetic alternatives [52]. Baculovirus-based insecticides typically require 4–5 days after application to demonstrate noticeable control results [86]. Although the delay is due to the time required for the virus to infect, replicate, and cause mortality in the target insects, studies on wild-type baculoviruses have shown that it is feasible to isolate and select strains with faster action, enhancing their effectiveness in pest control applications. Characterization of different geographical isolates of the same baculovirus or the study of genotypic variants within a single isolate has revealed great intraspecific variation within baculoviruses [87,88]. Small variations at the genomic level can result in significant phenotypic changes. Research has identified genetic variants of baculoviruses that exhibit differences in pathogenicity and speed of kill, allowing for targeted selection of faster-acting strains [89,90,91].

Selecting for high-speed-of-kill isolates, in contrast, may sometimes pose challenges in viral production. Baculoviruses are typically produced in vivo within insect larvae, but when larvae succumb to infection too quickly, viral yield can decrease due to insufficient time for replication and accumulation. This trade-off between speed of kill and production efficiency must be balanced to optimize both efficacy and productivity in IPM strategies.

Recent advancements in genetic engineering have paved the way for potential enhancements in baculovirus efficacy, with recombinant variants engineered to produce additional insecticidal proteins or altered hormone signaling pathways. These modifications aim to address the inherent limitations of baculoviruses, including their relatively slow speed of kill when compared to chemical insecticides. As reviewed by Moscardi et al [34], a variety of strategies have been explored. For example, eliminating certain nonessential viral genes can promote more rapid host mortality. Removal of the *ecdysteroid UDP-glucosyltransferase* (*egt*) gene, which normally modifies insect hormones and prevents their proper cellular uptake [92], leads to accelerated larval death. Introducing insect-derived genes that disrupt normal physiology is another approach. Incorporation of a diuretic hormone gene into the *Bombyx mori* NPV (BmNPV) accelerated mortality by approximately 20% [93], while expression of a pheromone biosynthesis activating neuropeptide (PBAN) gene fused to a bombyxin signal sequence in AcMNPV reduced *Trichoplusia ni* larval survival times by more than 20% [94]. Although attempts to utilize other insect hormone genes, such as those encoding eclosion hormone or prothoracicotropic hormone, did not yield significant improvements [95,96], the exploration of various insect hormones and enzymes remains promising.

In addition, recombinant baculoviruses expressing proteins like juvenile hormone esterase (JHE) were developed and evaluated primarily in the 1990s, demonstrating their capacity to rapidly arrest larval feeding and reduce crop damage [97]. These early studies showed the potential of such genetic modifications to accelerate baculovirus action. However, despite their promise, none of these recombinant viruses have reached the market to date. While strict regulatory frameworks and biosafety concerns associated with genetically modified organisms (GMOs) remain significant challenges, it has been suggested that the commercial discontinuation of recombinant baculovirus programs driven by pesticide-manufacturing companies in the USA was primarily driven by strategic considerations [98]. The rapid adoption of transgenic Bt crops, which offer fast-acting and broad-spectrum pest control, likely shifted industrial focus away from further development of recombinant baculoviruses, despite advances in regulatory clearance. As of now, no genetically modified baculovirus has received commercial approval, underscoring the need for continued research into their efficacy and safety to meet regulatory standards. Continued exploration of these genetic strategies could eventually transform the role of baculoviruses in sustainable pest management, provided current regulatory and safety barriers are addressed [32,99,100].

Moreover, the effectiveness and stability of baculoviruses are strongly influenced by environmental conditions, further shaping their practical application. Despite the fact that the polyhedrin protein that forms the outer shell of OBs provides some degree of resistance, factors such as UV radiation from sunlight and temperature and humidity fluctuations are known to degrade baculovirus OBs, thus limiting their persistence and efficacy in the environment [101,102]. Alkaline conditions in certain soils or on leaf surfaces can also impact viral stability [63,103]. As we previously mentioned, the biodegradability of baculoviruses is an environmental advantage. However, paradoxically, this characteristic can also be a disadvantage, as UV degradation may reduce their efficacy in the field. To mitigate these challenges, commercial formulations of baculoviruses are often enhanced with specific ingredients designed to protect the virus from environmental degradation. For example, formulations may include UV-blocking agents such as lignin, which can shield OBs from UV radiation, thus extending their efficacy in sunlight [104]. Additionally, certain microencapsulating materials like pregelatinized corn flour have been shown to improve the rainfastness of baculovirus formulations, ensuring that the virus remains active even after exposure to rain. These additives not only protect the virus from UV damage but also enhance its adherence to plant surfaces, reducing the impact of rainfall on its persistence [105,106]. Strategies aim to improve the overall performance of baculovirus-based insecticides in real-world field conditions.

Furthermore, while baculoviruses demonstrate excellent efficacy under controlled conditions, their field performance can sometimes fall short of immediate pest suppression requirements in high-pressure agricultural systems. This discrepancy stems from their intrinsic infection kinetics, which typically require 4–7 days to induce mortality [34,87]. Nonetheless, this limitation can be mitigated by timely application, integration with complementary approaches such as Bt formulations or selective insecticides [107,108], and improved formulations that enhance environmental persistence and uptake [105,106,109]. These combined strategies have shown promising results under field conditions [108,110] and contribute to narrowing the gap between experimental efficacy and field performance, thereby supporting the increasing inclusion of baculoviruses in integrated pest management (IPM) programs [34,107]. However, certain negative interactions between *B. thuringiensis* and baculoviruses have been reported [110]. Therefore, further studies should be conducted on this topic, especially considering the widespread use of transgenic crops based on Bt genes, in which baculovirus-based products are also applied.

Another significant limitation is the stability and storage of baculovirus-based products. These products need cold chain storage to maintain product viability, requiring refrigeration or freezing from production through to transport and storage [111]. This reliance on cold storage increases logistical costs by requiring specialized equipment and consistent temperature control, impacting final product pricing and hindering widespread adoption, particularly in regions lacking reliable refrigeration infrastructure. Research indicates that without cold storage, baculovirus products may experience a significant reduction in shelf life, diminishing their effectiveness in the field. In response, there is growing interest in developing stabilizing technologies—such as encapsulation and additive formulations—that reduce the need for cold storage and could make baculovirus-based products more accessible, especially in remote agricultural regions [112].

In summary, although the strengths of using baculoviruses as bioinsecticides are clear, further efforts are needed to improve the selection of natural baculovirus isolates with an appropriate host range and strong insecticidal characteristics, particularly in terms of speed of action and production efficiency. Additionally, the baculovirus industry must continue researching ways to enhance their stability, both during field application and storage, to maximize their effectiveness and commercial viability. Despite the broad diversity of baculovirus species identified, relatively few have successfully reached commercial formulations. The reasons for this are multifaceted, encompassing biological, ecological, and economic factors. The economic significance of the pest strongly influences the number of commercially available baculovirus-based products, with highly damaging pests like *H. armigera* motivating more extensive formulation development due to their widespread resistance and global economic impact [113]. Additionally, higher virulence or rapid killing speed, while beneficial for pest control, often reduces virus yield, creating a trade-off between production efficiency and commercial viability. Other challenges identified include narrow host specificity, formulation complexities (particularly UV stability), and high regulatory and production costs, all of which further restrict the development of diverse baculovirus formulations [114]. Nonetheless, an important advantage of baculoviruses is their excellent safety profile for human health and food crops. Regulatory agencies such as the EPA and OECD have concluded that baculoviruses pose no risk to consumers and do not require maximum residue limits. To date, no allergic reactions or adverse effects have been reported in humans, and even the presence of infected insect remains on plant surfaces does not compromise produce safety, as viral occlusion bodies degrade naturally and are non-infectious to vertebrates.

### 2.3. Baculovirus-Based Formulations

The development of baculovirus-based insecticides involves converting raw viral material—OBs—into stable, effective, and marketable products suitable for field application. A formulation integrates the active ingredient with carriers, adjuvants, and, when beneficial, innovative encapsulation technologies, all while adhering to production, handling, and regulatory requirements [109,115]. Although the principles resemble those of other microbial insecticides, baculoviruses demand particular attention to environmental stability, UV protection, and the particulate nature of their infectious units [36,116,117,118].

#### 2.3.1. Production and Quality Control of the Active Ingredient

The fundamental requirement for any baculovirus-based bioinsecticide is to produce a reliable product in adequate quantities, of suitable quality, and at an affordable cost [119,120]. Baculoviruses, like all viruses, require living host cells for replication [52,120]. These cells may be derived from intact insects (in vivo production) or tissue culture systems (in vitro production). Regardless of the production method, it is crucial to generate large quantities of viable OBs—the robust protein matrices that protect the virions—under consistent and controlled conditions [119,121].

In in vivo production, healthy and disease-free insect hosts must be maintained to ensure high yields and stable product quality [122,123]. A dependable insect rearing system necessitates rigorous management of critical parameters—including temperature, humidity, diet, and sanitation—to substantially reduce the risk of introducing unwanted pathogens [124]. Host selection can be further complicated by the existence of narrow or broad baculovirus host ranges, and certain isolates may only replicate in a single insect species, whereas others have a wider host range [36,119]. Properly separating “clean” (host rearing) and “infected” (virus production) areas prevents unwanted cross-contamination and ensures that the original culture remains uninfected by covert pathogens [122,124].

Once a suitable host–virus system is identified, parameters such as the initial inoculation dose, larval instar, incubation conditions, and harvest timing are optimized to maximize OB yield [125,126,127]. For nucleopolyhedroviruses (NPVs), yields often range between 10^9^ and 5 × 10^9^ OBs per larva, whereas GVs can reach up to 10^11^ OBs per larva [121,128]. Certain GVs, such as CpGV or Cryptophlebia leucotreta granulovirus (CrleGV, *Betabaculovirus cryleucotretae*), have shown even higher yields in some host species [128,129,130]. Higher larval density can improve production at the container level but sometimes reduces the obtained yield per larva [130,131]. In addition, harvesting infected larvae at different stages (dead vs. moribund) can influence the subsequent biological activity of the OBs [132,133,134].

Despite the practical simplicity and relative low cost of in vivo systems, scaling up for large-scale field applications can be challenging [135]. In contrast, in vitro production in insect cell culture provides a more controllable and potentially automatable system for mass production of high-quality OBs without high labor costs [136,137,138]. However, to date, no in vitro system has been fully commercialized because of limitations in cost-effective media, the fragility and high oxygen demand of insect cells in large bioreactors, and the risks of accumulating defective viral phenotypes that reduce infectivity [111,120,138,139]. Moreover, current in vitro systems are mainly optimized for the infection of Sf9 cells with AcMNPV, while there is a lack of susceptible cell lines for many baculoviruses of biocontrol interest, further limiting their applicability beyond model systems. In addition, in vitro production remains expensive and technically demanding [140]. These technical and economic barriers need to be resolved before in vitro production methods become widely feasible, especially for mainstream agricultural markets requiring high-volume production [138].

Quality control is central to ensuring the consistent efficacy and safety of baculovirus-based products [141,142]. For the virus itself, a purified reference stock is essential [119,122]. This stock should be genetically characterized, cryopreserved, and stored in culture collections for consistency and traceability. The production process for baculovirus-based products is relatively straightforward: the virus propagates efficiently when ingested by a susceptible host provided that optimal conditions (adequate temperature, humidity, and nutrition) are met. However, comprehensive record keeping of all production parameters is crucial. Comprehensive records—detailing environmental conditions, larval rearing metrics (such as weights at infection and at harvest), diet batch quality, yield assessments (via light microscopy, bioassays, and DNA profiling), and contamination monitoring—should be meticulously maintained and routinely reviewed by an independent entity. This systematic record keeping ensures that any subtle, yet potentially critical, deviations are promptly identified and corrected, thereby maintaining product quality and consistency [143]. After obtaining large quantities of viable OBs under these controlled conditions, the active ingredient can be further processed into a dry format. Dry formulations can be prepared using freeze-drying and air-drying technologies, each with distinct advantages in terms of stability and application potential [144,145]. This drying process preserves the integrity and potency of the OBs prior to their incorporation into the final biopesticide formulations.

Spectrophotometry, qPCR, hemocytometer-based OB counting under phase-contrast microscopy, and bioassays measuring infectivity (e.g., median lethal dose (LD_50_) or median lethal concentration (LC_50_)) have traditionally been employed as standard methods for assessing product potency, including the quantification of infectious viral content. However, spectrophotometry has previously been shown to overestimate occlusion bodies, particularly in granuloviruses [146]. Counting OBs using a Neubauer hemocytometer remains the most common approach for nucleopolyhedroviruses due to its practicality and reliability [147,148]. In contrast, for granuloviruses—whose occlusion bodies are significantly smaller and less visible under conventional light microscopy—qPCR presents a notable advantage, as it is unaffected by contamination from biological or non-biological debris, which often interferes with microscopic and spectrophotometric methods [149,150]. In addition, molecular techniques such as PCR and gene sequencing are routinely employed to verify the identity of the viral strain and detect covert infections [151]. To ensure the absence of undesired chemical contaminants, regulatory frameworks also recommend routine testing for residues via chemical analyses and bioassays.

Microbial contaminants—primarily bacteria, yeasts, and occasional fungi—are managed through a combination of factors, including selecting appropriate harvest times to prevent excessive bacterial proliferation [134,152] and employing downstream processes such as freeze-drying [144]. Aerobic microbial contaminants in OB suspensions typically range from 10^7^ to 10^8^ CFU/mL based on standard culturing methods [83,152,153,154], with bacterial counts increasing rapidly after larval death due to cadaver colonization [134,152]. However, yeasts and molds are generally present in lower concentrations, ranging from 10^3^ to 10^5^ CFU/mL [83,152,154]. These contaminants are considered a key quality control issue, as regulatory agencies impose limits on their permissible levels in virus-based insecticides [119,121]. According to the OECD guidelines, acceptable limits for microbial contaminants in baculovirus-based formulations are set at or below 5 × 10^8^ CFU/g for total aerobic mesophilic microorganisms in powder formulations, with additional restrictions on specific microbial groups, including yeasts and molds (≤10^5^ CFU/g), coliforms (≤10^3^ CFU/g), and the absence of *Escherichia coli* and *Salmonella* spp. [155]. Hence, a combination of controlled larval rearing, precise infection protocols, and standardized downstream processing can yield high-titer formulations that meet regulatory and commercial standards for microbial control agents [141,153,156].

Overall, both in vivo and in vitro production systems continue to be developed and optimized for different baculovirus–host combinations. While in vivo production is currently the only economically viable means of large-scale baculovirus propagation [52,123,138], significant research efforts persist in in vitro methods, given the potential advantages of process automation and uniform product quality [136,137]. Recent studies, such as Klafke et al. [157], have demonstrated promising advances in this area. Specifically, they reported the successful large-scale production of SfMNPV in bioreactors using optimized culture conditions, achieving high OB yields and demonstrating the technical feasibility of commercial in vitro OB production. Regardless of the approach, a rigorous quality control program—encompassing host insect health, inoculum characterization, OB quantification, infectivity assays, and contaminant limits—remains fundamental to delivering effective baculovirus-based bioinsecticides [135,141]. Additionally, genetic characterization and microscopic verification are essential to confirm that the virus in each production batch remains genetically stable and structurally intact [158]. These steps help ensure fidelity to the original reference isolate, preventing the accumulation of unwanted genotypic variants that could compromise efficacy or safety [35].

#### 2.3.2. From Raw Virus to Formulated Product: Carriers, Adjuvants, and Encapsulation

Once a quality-controlled active ingredient is obtained, the next challenge is to develop a formulation that meets practical field requirements. Baculovirus-based bioinsecticides must balance stability, efficacy, and ease of application to ensure successful field performance. Several key formulation components contribute to this goal, including inert materials that support viral OBs, additives that enhance their biological and physical properties, and advanced technologies designed to extend their persistence in the field.

The use of inert carriers plays a crucial role in maintaining OB stability, facilitating their uniform dispersion, and controlling their release kinetics in baculovirus-based formulations. Depending on the formulation type, these carriers can be either mineral-based (e.g., diatomite, clay, silica, kaolin) or liquid-based (e.g., water or oil emulsions) [159,160,161]. In dry formulations, which rely predominantly on solid carriers, these materials not only protect microbial biopesticides from desiccation and other environmental stressors but also enhance overall viability by shielding OBs from adverse conditions and extending shelf life. Common solid carriers used in these formulations include alginate, lignite, kaolinite, montmorillonite, peat, pyrophyllite, press mud, sawdust, turf, talc, vermiculite, and zeolite [162]. In addition, the incorporation of binders, dispersants, wetting agents, and other stabilizing additives further improves product consistency and ease of application. Nevertheless, despite these advancements, formulating baculoviruses in dry formats still presents challenges regarding OB stability, dispersibility, and field persistence, necessitating careful optimization of carrier selection and processing conditions [145].

Beyond carriers, adjuvants are incorporated into baculovirus formulations to enhance their physical and biological properties. These include surfactants, UV protectants, stickers, and feeding stimulants, each contributing to increased product effectiveness [104,163]. Surfactants and wetting agents play a crucial role in biopesticide formulations by enhancing their emulsifying, dispersing, spreading, sticking, and wetting properties [81]. These compounds reduce surface tension, ensuring a more uniform distribution of the active ingredient across plant surfaces. By improving coverage and adhesion, surfactants help optimize the effectiveness of baculovirus applications, minimizing run-off and increasing the likelihood of pest contact and ingestion. UV protectants, such as lignin derivates and experimentally tested compounds like optical brighteners, mitigate photodegradation—a major limitation for baculovirus persistence in open-field conditions [117,164,165]. Although optical brighteners have demonstrated efficacy in reducing LC_50_ and time-to-lethality (LT_50_) values in laboratory settings [166], they are not currently included in any commercially registered formulations. Stickers and binders, including cellulose derivatives, molasses, and vegetable gums, improve adhesion to plant surfaces and increase rainfastness, reducing product loss due to environmental factors [167]. Feeding stimulants, such as molasses, enhance virus ingestion by target pests, leading to higher infection rates and improved control efficacy [144]. While these adjuvants improve baculovirus efficacy, their interactions with OBs must be carefully considered. Some surfactants can disrupt OB integrity, while excessive amounts of stickers may interfere with viral bioavailability. Additionally, optical brighteners have been associated with reduced LC_50_ and LT_50_, necessitating precise calibration of their inclusion in formulations [168].

In addition to the role of adjuvants in improving formulation performance, further advancements in baculovirus stability and persistence can be achieved through encapsulation technologies, which provide an additional layer of protection against environmental degradation. Encapsulation technologies offer an additional strategy for enhancing baculovirus stability and persistence [80,104]. By embedding OBs within protective polymeric matrices, such as lignin or methacrylic acid polymers, encapsulation improves photostability, reduces wash-off due to rain or irrigation, and can even enhance insecticidal activity compared to unformulated viruses [80,169]. Various encapsulation approaches exist: lignin-based encapsulation leverages natural UV protective properties, while polymer-based microencapsulation allows for controlled release [170], gradually exposing the virus to the target pest over time [104]. For example, a *Spodoptera frugiperda* nucleopolyhedrovirus (*Alphabaculovirus spofrugiperdae*, SfMNPV) was successfully microencapsulated with methacrylic acid polymer using an oil-in-oil emulsion solvent evaporation method, improving viral photostability without compromising insecticidal activity [80]. A notable advantage of this pH-sensitive polymer is its mucoadhesive property, which increases the absorption of drugs formulated with this material [171]. Methacrylic acid polymer is also hydrophobic with a high affinity for lipid membranes, and its microparticles undergo pH-dependent swelling, transitioning from a solid to a gel state, a characteristic that enhances adhesion to mucous membranes [172]. Additionally, Arthurs et al. found that the insecticidal activity of CpGV was significantly higher when formulated with lignin (92–93.6% mortality) compared to an unformulated virus (67.2%) under simulated solar irradiation. This improvement highlights the UV protective effect of lignin, which helps maintain viral activity despite exposure to potentially damaging sunlight [104]. However, once again, there is a gap between experimental development and practical application. Barriers such as high costs may explain why this technique is not widely used today.

Recent innovations have narrowed the performance gap between biopesticides and conventional chemical insecticides, delivering enhanced field effectiveness while preserving environmental integrity [173]. Nonetheless, scaling up the production of encapsulated formulations remains a significant hurdle, as cost-effective manufacturing strategies must be developed to maintain optimal virus bioavailability.

#### 2.3.3. Formulation Types and Application Formats

The integration of optimized viral production, quality control, carrier selection, and adjuvant incorporation culminates in a range of final formulations tailored to agricultural needs. Baculovirus commercial products commonly appear as wettable powders (WPs) or suspension concentrates (SCs) suitable for conventional spraying after dilution with water [121]. In SC format, the active ingredient is dispersed in an aqueous solution. The main advantages of this format are its ease of application and the absence of dust. In contrast, solid forms, such as wettable powders, granules, or water-dispersible granules (WDGs), streamline transport, storage, and dosing, and meet the logistical demands of large-scale field operations. More advanced formats, including oil dispersions (ODs), microencapsulated suspensions (CSs), microemulsions (MEs), or concentrated aqueous emulsions (EWs), can be adapted to overcome particular environmental constraints or improve compatibility with existing equipment [115,174,175].

By addressing the technical challenges of converting raw viral material into refined, field-ready formulations—incorporating carriers, adjuvants, and state-of-the-art encapsulation techniques—we lay a practical foundation for employing baculoviruses as effective pest management agents. The following section offers a global perspective on baculovirus applications, outlining key lepidopteran targets, current market dynamics, and emerging trends that define their role in sustainable agriculture.

## 3. Global Overview of Baculoviruses in Pest Control: Key Lepidopteran Targets, Market Dynamics, and Trends

### 3.1. Principal Lepidopteran Pests Controlled by Baculoviruses

The following section focuses on the most economically damaging lepidopteran pests and the baculovirus-based products currently available to mitigate their impact. The main lepidopteran pests causing the greatest economic losses in crops include *C. pomonella* [176], *Helicoverpa* spp. [177,178], *Mamestra brassicae* [179], *Plutella xylostella* [180], and *Spodoptera* spp. [181,182]. Managing these pests is crucial due to their widespread impact and the economic losses they cause. Data on their main host plants and global distribution emphasize the necessity for robust pest management strategies. While the tables in this section provide information on commercial products, target pests, and OB dose per hectare, data on cost per hectare are not included due to the lack of standardized, publicly available sources and high variability across regions and formulations.

#### 3.1.1. *Cydia pomonella*

*C. pomonella* (L.) (Lepidoptera: Tortricidae), commonly known as the codling moth, it is a major pest of pomefruit crops such as apples and pears (Table 1). It has caused substantial losses globally, particularly in regions like Asia, eastern Australia, and North America, making it a critical target for improved pest control methods [176,183]. Products based on the CpGV have been successfully used since the late 1980s for the control of this pest, mainly based on the CpGV-M original isolate from Mexico [37,184]. The intensive use of this isolate with low genetic diversity may be the origin of the first occurrence of resistance described for baculoviruses, and overcoming this resistance has become a matter of crucial importance for the baculovirus industry [185,186,187,188]. Nowadays, several baculovirus-based products for this pest are registered worldwide. Products like Carpovirusine and Carpovirusine evo2, registered by UPL Europe Ltd. in Europe and South America, utilize a CpGV isolate effective against *C. pomonella* populations resistant to the CpGV-M as the active ingredient. Similarly, MADEX, produced by Andermatt and registered in multiple continents, contains CpGV isolates at a higher concentration and comes in variants like Madex Twin, Max, Top, and Hp, each tailored for specific regions or additional pests, like *Grapholita molesta*. CYD-X, another widely used product produced by Certis, is registered globally and offers flexible application rates depending on tree size (Table 1). All these products are formulated as SCs and are designed to target different larval stages of the pest, with storage recommendations at 4 °C. These baculovirus-based products provide environmentally friendly and species-specific options for controlling *C. pomonella*, reducing reliance on chemical insecticides and mitigating the economic impact of this pervasive pest.

#### 3.1.2. *Helicoverpa armigera*

*Helicoverpa* spp., *H. armigera* (Hübner) (Lepidoptera: Noctuidae), commonly known as the cotton bollworm, is one of the most damaging and widespread polyphagous lepidopteran pests, affecting a wide range of crops globally: Anacardiaceae, Fabaceae, Asteraceae, Pinacceae, Rutaceae, Malvaceae, Brassicaceae, Moraceae, Cucurbitaceae, Rosaceae, Solanaceae, and Poaceae [177,189,190]. This pest was restricted to Europe, Africa, Asia, and Australasia, causing losses estimated to exceed USD 2 billion, until its introduction in Central and South America in the early 21st century [191].

Since its introduction into Brazil during December 2012 and January 2013, it has caused devastating damage to crops such as soybeans and cotton [192]. Farmers faced significant challenges due to its resistance to both chemical insecticides and Bt strains [113,193]. The lack of effective control measures led to the declaration of an emergency state by the Brazilian Ministry of Agriculture and the approval of several products, both chemical and biological. Notably, Agbitech’s Armigen, based on the Helicoverpa armigera nucleopolyhedrovirus (HearNPV, *Alphabaculovirus helarmigerae*), was rapidly registered due to the company’s previous expertise in managing *H. zea* in Australia. The product demonstrated high efficacy, leading to widespread adoption by farmers [194]. This event marked a pivotal moment in Brazil’s biocontrol landscape, prompting increased demand for biocontrol products and encouraging regulatory authorities to streamline registration processes for such agents [192,195].

This has necessitated innovative approaches in pest management. The urgent need for effective control methods for *H. armigera* has led to the development and registration of a wide range of nucleopolyhedrovirus-based products. The proliferation of these products is partly due to the pest’s significant economic impact and its rapid spread across continents, which has prompted regulatory authorities to expedite the approval of biocontrol agents. Products like Helicovex by Andermatt Biocontrol utilize the HearNPV and have become widely adopted due to their effectiveness. Another kind of product, namely Surtivo Soja [196], whose active ingredient is a mixture of the HearNPV and the Chrysodeixis includens nucleopolyhedrovirus (ChinNPV, *Alphabaculovirus chrincludentis*) for the control of multiple pests including *Chrysodeixis includens* and *H. armigera*, reflects a strategic approach to pest management by providing broader-spectrum control.

Products based on the HearNPV have been registered not only in Brazil but also in other regions such as the US, Europe, or China. Given that *H. armigera* is such a significant pest, it is often also included as a target within the spectrum of broad-spectrum baculovirus-based products. Products like VPN Ultra, Surtivo Plus, and Surtivo Soja utilize different mixtures of viruses. Additionally, one product from Unioasis broadens the spectrum further by combining a synthetic insecticide with a nucleopolyhedrovirus [197] (Table 2). Surtivo Soja controls *C. includens*, *H. armigera*, *H. zea*, *Chloridea virescens*, and *Heliothis virescens*, whereas Surtivo Plus expands its spectrum to include *Spodoptera eridania* and *S. frugiperda* by incorporating four different nucleopolyhedroviruses. VPN Ultra is designed for wide-spectrum efficacy and targets numerous defoliating and boring larvae in crops such as cotton, broccoli, chili, tomato, tobacco, soybean, pineapple, melon, cereals, legumes, asparagus, okra, cabbage, watermelon, and ornamental flowers. It is effective against genera including *Helicoverpa*, *Spodoptera*, *Trichoplusia*, *Diaphania*, and others, and is also used in forestry crops like pine. Others like Diplomata Evo target *H. armigera* and *C. includens*, using a mixture of the HearNPV and ChinNPV. The significant number of baculovirus products for *H. armigera* underscores the agricultural community’s response to the pest’s adaptability and resistance. Farmers’ adoption of these biocontrol products has been a turning point, leading to a shift in pest management strategies and prompting regulatory authorities to favor these substances [113].

#### 3.1.3. *Mamestra brassicae*

*M. brassicae* (L.) (Lepidoptera: Tortricidae), or the cabbage moth, is another pest causing significant damage to horticultural crops due to its extensive dietary range: Asteraceae, Brassicaceae, Chenopodiaceae, Fabaceae, Liliaceae, Solanaceae, and Poaceae [179,198,199]. This species is predominantly distributed throughout Asia and Europe, further emphasizing its potential impact on agricultural production in these regions [198]. The larvae feed on a variety of plants, necessitating robust control strategies. The *Mamestra brassicae* multiple nucleopolyhedrovirus (MbMNPV, *Alphabaculovirus mabrassicae*) has the particularity of having a relatively broad host range [200]. In Europe, the insecticide Mamestrin^®^ based on the MbMNPV was commercialized by Natural Plant Protection, but the use of this virus is not currently approved under European regulations. Another MbMNPV-based biocontrol product named Envivo^®^ SC, produced by Point Andina, offers control of other lepidopteran pests [201]. At this moment, this product is commercialized only in some countries of South America where *M. brassicae* is not present.

#### 3.1.4. *Plutella xylostella*

*P. xylostella* (L.) (Lepidoptera: Plutellidae), the diamondback moth, a major pest of *Brassicaceae* crops such as cabbage, cauliflower, and other cruciferous species distributed worldwide, has developed resistance to nearly all commercial chemical insecticides, leading to increased economic losses, particularly in China [180,202]. Additionally, field-evolved resistance to Bt subsp. *kurstaki* (Btk)—a main component of commercial products like Dipel—has been documented, notably reducing its efficacy in populations repeatedly treated with Btk-based formulations in the field. For instance, significant resistance was observed in populations from farms in Hawaii after frequent applications, with mortality dropping to as low as 34–35% compared to over 90% in susceptible laboratory colonies [203]. This challenge has necessitated the development of baculovirus-based products targeting *P. xylostella*. Products like Plutex, registered in North America by Andermatt, use the *Plutella xylostella* granulovirus (PxGV, *Betabaculovirus pluxylostellae*) as the active ingredient [204]. In addition to these species-specific products, several broad-spectrum options also target *P. xylostella* among other pests. On one hand, some broad-spectrum solutions combine baculoviruses with Bt, such as SeNPV + Bt (Wuhan Chuqiang Biological Technology Co., Ltd., 2022) and Bypel 1 (Unioasis). On the other hand, products like Vpn Ultra (Agricola El Sol, n.d.), Lepigen (Agbitech), and Envivo^®^ SC rely solely on baculovirus isolates to achieve broader control (Table 3). Lepigen contains a broad-host-range nucleopolyhedrovirus as its active ingredient; however, its registered use is limited to the control of *P. xylostella*. Envivo^®^ SC, in particular, targets a wide range of pests including *C. pomonella*, *C. molesta*, *Proeulia auraria*, *P. xylostella*, *Tuta absoluta*, *Phthorimaea operculella*, *Heliothis zea*, *Rachiplusia ou*, *S. frugiperda*, *Agrotis ipsilon*, and *Dalaca* sp.

#### 3.1.5. *Spodoptera* spp.

The genus *Spodoptera* includes some of the most significant agricultural pests worldwide, known for their high adaptability and resistance to control measures. Their impact on global agriculture has made them a central focus of pest management research. Species such as *S. frugiperda* and *Spodoptera littoralis* rank among the top 15 most resistant arthropods worldwide [205]. Each species presents unique challenges and distribution patterns: *S. exigua* has a global presence [206,207]; *S. litura* is widely distributed across South and East Asia and Oceania, particularly in tropical and subtropical regions where there are no, or few, frost days each year [208]; *S. littoralis* is found in Africa, Southern Europe, and West Asia [209]; and *S. frugiperda* is native to the Americas but has rapidly expanded its range to Africa, Europe, Asia, and Oceania in recent years, and was detected as far as Australia by 2020 [210,211,212]. In Europe, its arrival has heightened concerns due to quarantine regulations, limited previous exposure, and the pest’s known capacity to inflict severe yield reductions.

*S. frugiperda* (J.E. Smith) (Lepidoptera: Noctuidae), commonly known as the fall armyworm, infests more than 353 host plants spanning 76 botanical families—including Fabaceae, Cucurbitaceae, Malvaceae, Poaceae, Zingiberaceae, Convolvulaceae, Musaceae, Brassicaceae, Chenopodiaceae, and Solanaceae—leading to substantial economic losses and significant threats to global food security [178,213,214]. In Brazil, populations of *S. frugiperda* have developed resistance to synthetic insecticides and chitin biosynthesis inhibitors, indicating an urgent need for sustainable, integrative management strategies [215]. In response to these challenges, baculovirus-based products targeting *S. frugiperda*—primarily formulations of the SfMNPV—have been developed and are widely registered in South America. Commercial products like Vircontrol-Sf [216], Cartugen [217], Spodovir Plus [218], and Fawligen [219] reflect the global significance of *S. frugiperda* and the need for effective control measures (Table 4). Spodovir Plus controls both *Spodoptera frugiperda* and *Spodoptera cosmioides*, broadening its potential application in areas where these species co-occur. Some broad-spectrum formulations, such as Surtivo^®^ Plus [220] and Vpn Ultra, combine different nucleopolyhedroviruses to target a wider range of pests, while others (e.g., Envivo^®^ SC) employ broad-host-range viruses as active ingredients. Interestingly, despite differing brand identities, many commercial SfMNPV-based products rely on identical or closely related isolates [221]. These solutions are essential in IPM, offering environmentally sustainable solutions and helping to mitigate resistance development [215]. The economic damage caused by *S. frugiperda* and its resistance to chemical insecticides justify the extensive list of baculovirus products designed for its control.

Finally, *S. littoralis* (Boisduval) (Lepidoptera:Noctuidae), the Egyptian cotton leafworm, targets crops belonging to more than 40 families, including Lithomytrus, Malvaceae, Apiaceae, Asteraceae, Brassicaceae, Chenopodiaceae, Convolvulaceae, Cucurbitaceae, Fabaceae, Lauraceae, Liliaceae, Moraceae, Musaceae, Punicaceae, Rosaceae, Rubiaceae, Rutaceae, Solanaceae, Theaceae, and Tiliaceae [182,222]. Found in North Africa, Southern Europe, and West Asia [209], it poses a significant threat to agriculture. Recognizing its negative impact on productivity and biodiversity, the European Union’s Directive 2000/29/EC classifies *S. littoralis* as a harmful organism. Baculovirus-based products, such as Littovir^®^ by Andermatt Biocontrol, employ the *Spodoptera littoralis* nucleopolyhedrovirus (SpliNPV, *Alphabaculovirus splittoralis*) to achieve species-specific control. The few commercial products available for controlling *S. littoralis* point to the need for additional research and development to bolster IPM strategies, to cut back on chemical insecticide use, and to lessen environmental impact.

The development and registration of baculovirus-based products reflect the agricultural community’s response to the challenges posed by these major lepidopteran pests. The varying number of products available for each pest correlates with the economic impact, geographical spread, and resistance levels exhibited by the pests. For instance, the multitude of products for *H. armigera* is a direct response to its widespread resistance and significant damage to crops globally, necessitating diverse and effective control options. These biocontrol agents offer environmentally friendly alternatives to chemical insecticides, playing a crucial role in sustainable agriculture and IPM.

### 3.2. Baculovirus Market Expansion and Trends

#### 3.2.1. Leading Companies in the Sector

**Figure 3:**
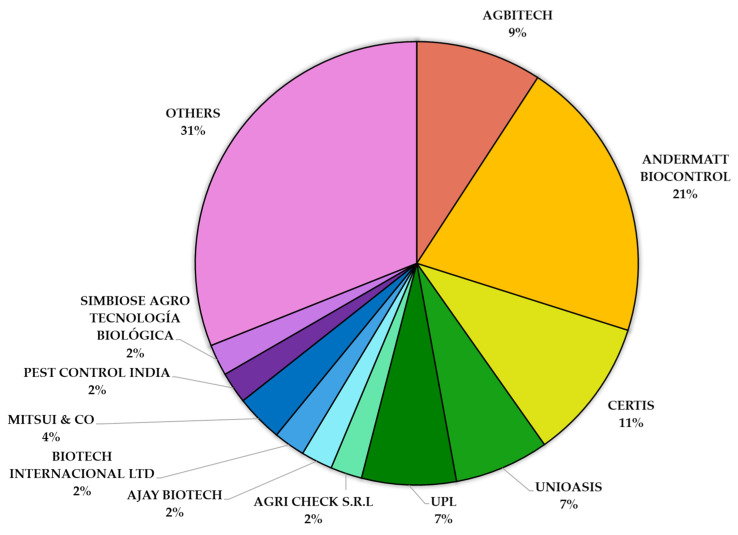
Global percentage distribution of baculovirus-based microbial insecticides registered by company. Data compiled by authors from publicly available sources and product registration records.

The global market for bio-based pest control solutions, including baculovirus-based products, is expanding, spurred by the demand for chemical-free agriculture and the growing organic food sector [223]. Major industry players such as Syngenta AG, BASF SE, Bayer, and UPL have entered this dynamic landscape, alongside specialized biocontrol companies like Andermatt Biocontrol, Certis, AgBiTech, Koppert, and Unioasis. Among them, Andermatt Biocontrol leads the market in terms of the number of baculovirus-based products (21%), followed by Certis (11%), AgBiTech (9%), and Unioasis, each of which account for 7% of the market (Figure 3).

Andermatt Biocontrol’s leadership in this sector is largely due to its early entry into the baculovirus market. Founded in 1988 in Switzerland, the company was one of the first to commercialize baculovirus-based bioinsecticides, with Madex (CpGV) being one of its flagship products. This long-established presence has given Andermatt a particularly strong foothold in Europe, where it is the biggest distributor of baculovirus based bioinsecticides.

In contrast, AgBiTech has emerged as a rapidly growing competitor, despite being a relatively newer player. Since being founded in the early 2000s in Australia, the company has significantly expanded its portfolio, starting with Vivus Gold (HaNPV) and later incorporating multiple baculovirus-based solutions targeting key agricultural pests. As explained before, the rapid growth of this company is directly linked to the phytosanitary crisis caused by the introduction of *H. armigera* in Brazil and the success of its product Vivus Gold, which was already being marketed in Australia, during that period. Its fast-paced growth reflects the increasing global demand for bio-based alternatives to synthetic insecticides.

Additionally, a substantial portion (25%) of the market consists of numerous smaller companies—each holding less than 5% of the global share—collectively grouped under ‘Others.’ Companies such as Mitsui & Co (4%), Biotech Internacional Ltd. (2%), Ajay Biotech (2%), Pest Control India (2%), Simbiose Agro Tecnología Biológica (2%), and Agri Check S.R.L. (2%) contribute to the remaining market share (Figure 3).

#### 3.2.2. Global Disparities and Emerging Trends in Baculovirus Bioinsecticides

**Figure 4:**
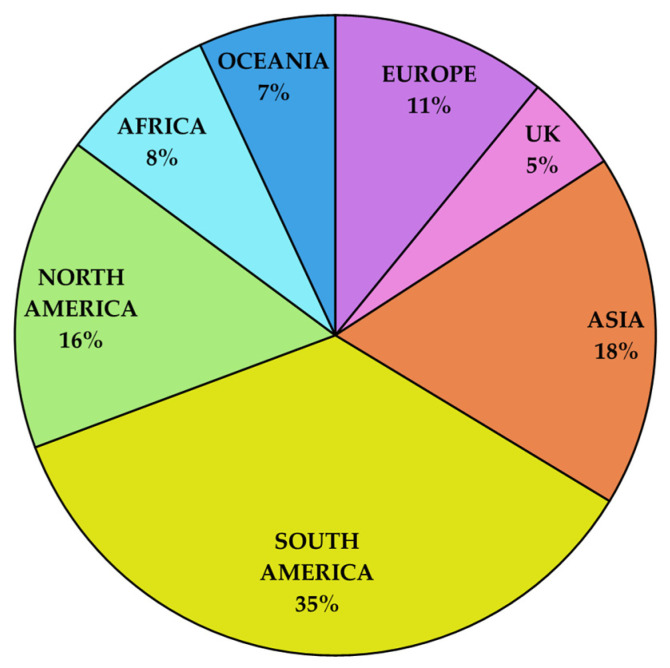
Global distribution of baculovirus-based product registrations by region (%). Data compiled by authors from publicly available sources and product registration records.

South America leads the global market with the highest percentage of registered baculovirus-based products, as illustrated in Figure 4. This dominance reflects the region’s supportive regulatory environment and focus on biological pest management solutions, particularly in Brazil. Comparatively, Asia accounts for 18% of registrations, driven by its growing bioinsecticide adoption but with a preference for mixed formulations (e.g., baculoviruses combined with Bt or chemical insecticides). North America and Europe contribute 16% and 11%, respectively, although differing regulatory landscapes influence product diversity and market growth.

The current global scenario of baculovirus-based bioinsecticides reveals a market shaped by complex interactions between regional pest challenges, regulatory environments, and evolving agricultural practices. One of the most striking aspects is the substantial proportion of registered products in South America, where high pest pressures—especially from species like *S. frugiperda*, *H. armigera* and *C. includens*—have driven demand for effective alternatives to conventional chemical pesticides. This urgency, combined with more flexible registration frameworks, notably in Brazil, has fostered a rapidly growing sector of biologically based pest control. As a result, South America is not only home to numerous specialized formulations targeting priority pests but also to an emerging category of broad-spectrum products that combine multiple baculovirus isolates to manage complex pest populations with fewer treatments.

In contrast, other regions present a different dynamic. Asia, influenced by intensifying agriculture and pest resistance issues, tends to favor integrated strategies where baculoviruses are used alongside Bt or other agents. This approach underscores a willingness to adopt versatile solutions but also highlights regulatory and market conditions that currently favor mixed formulations over purely baculovirus-based broad-spectrum products.

North America, benefiting from relatively streamlined regulatory procedures, has commercialized numerous baculovirus products, yet these tend to remain more target-specific, possibly reflecting a different balance of pest pressures, the availability of genetically modified (GM) crops, and established IPM practices that already reduce reliance on a single type of solution.

Europe, despite strong commitments to sustainability and the promotion of IPM, has fewer registered baculovirus products. Here, stricter regulatory standards, coupled with comparatively lower pest pressure, have limited the range of available formulations. The result is a market that still favors specificity and compliance with rigorous safety standards over rapid diversification. Smaller but emerging markets like Africa and Oceania face their own constraints—whether in terms of regulatory immaturity or highly specialized farming systems. These conditions can restrict product variety but also create niches for targeted solutions, as seen with certain products focused on local key pests in Oceania.

**Figure 5:**
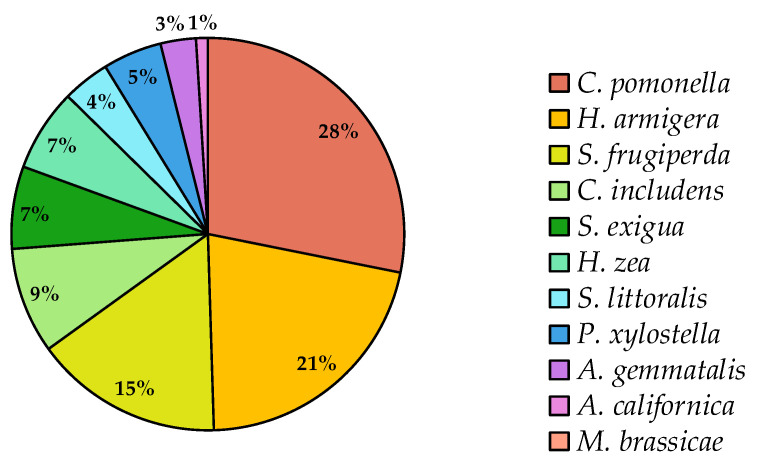
Availability of baculovirus-based products for pest control by target species worldwide. Data compiled by authors from publicly available sources and product registration records.

Taken together, these patterns explain why some pests have drawn more attention than others. Species such as *C. pomonella* and *H. armigera*, with their global economic importance and established resistance problems, have spurred significant investment in tailored baculovirus-based solutions. These species account for nearly half of the market share (Figure 5). By contrast, other equally damaging pests have not attracted the same level of research or product development, often due to narrower geographic distributions, smaller market potential, or more complex management challenges.

Baculovirus products are predominantly formulated as SCs, which account for 88% of the market, while solid formats (WPor, water-dispersible granules (WGs)) comprise the remaining 12%. This preference for liquid formulations may be attributed to their ease of handling, improved application efficiency, and better compatibility with conventional spraying equipment, making them more convenient for large-scale agricultural use.

Due to the inherent properties of baculoviruses discussed earlier, most baculovirus-based products available on the market exhibit high specificity. However, the recent expansion of broad-spectrum solutions highlights the practical adaptability of baculoviruses. These products address the challenges of diverse pest complexes in varying field conditions, further enhancing their role in IPM strategies. To enhance efficacy, some formulations combine baculoviruses with chemical insecticides, aiming to leverage the specificity of baculoviruses while benefiting from the rapid knockdown action of chemical agents. However, the overall safety of such formulations depends on the specific chemical used, as the inclusion of a conventional insecticide may alter the environmental and toxicological profile of the final product. For instance, AgBiTech recommends applying their Cartugen (SfMNPV) product together with a short-residual chemical insecticide like methomyl to quickly lower pest pressure, allowing the baculovirus to provide sustained control over time. These integrated approaches demonstrate the versatility of baculoviruses in pest control while supporting the long-term sustainability of IPM strategies. However, these approaches require careful planning to avoid adverse interactions and ensure ecological compatibility.

Future trends suggest that the market will progressively favor broad-spectrum formulations. While the core strength of baculoviruses lies in their host specificity, the practical needs of agriculture—managing pest complexes efficiently, addressing resistance issues, and reducing the number of field applications—are fueling interest in mixtures of multiple isolates and integrated products. Advances in formulation technology, including encapsulation, UV protectants, and the widespread adoption of SC formulations, could further expand product usability and shelf life, overcoming one of the current weaknesses: the dependency on cold storage and environmental susceptibility.

Ultimately, the trajectory of baculovirus-based bioinsecticides will depend on the continued harmonization of regulatory frameworks, ongoing research to broaden their applicability, and increasing farmer awareness and education. As markets mature, we are likely to see a more balanced portfolio of products, with highly specific solutions coexisting alongside broad-spectrum formulations designed for regions confronting diverse pest complexes. This diversification, supported by improved formulation technologies, integration with chemical or biological agents, and possibly aided by carefully regulated biotechnological innovations, suggests a future in which baculovirus-based solutions become integral components of sustainable, globally responsive pest management strategies.

## 4. Regulatory Frameworks for Biopesticides: A Global Comparison with Focus on Baculovirus-Based Products

The global landscape for biopesticide regulation is characterized by significant diversity and complexity, reflecting the unique priorities and challenges faced by different regions. In this section, we will analyze the legislation in the most significant regions for biopesticides, where these products have the largest markets and play a crucial role in pest management strategies.

### 4.1. United States

In the United States, the Federal Integrated Pest Management Coordinating Committee (FIPMCC) promotes and coordinates IPM practices. The National IPM Road Map, updated in 2018, aims to increase the adoption, implementation, and efficiency of safe, economical pest management practices. The Environmental Protection Agency (EPA) regulates pesticide registration, requiring all pesticides sold or distributed in the US to be registered. The Federal Insecticide, Fungicide, and Rodenticide Act (FIFRA) governs pesticide use, supplemented by the Pesticide Registration Improvement Act (PRIA), last updated in 2022 (PRIA5). The recent regulation revised fees and review times and included provisions for farm worker protections and bilingual labeling (English–Spanish). The EPA encourages the development and use of biopesticides, requiring less data and shorter registration times than for chemical pesticides. Section 18 of FIFRA allows the EPA to grant emergency exemptions for unregistered pesticide uses [156]. These exemptions authorize temporary use of pesticides in emergency situations where no registered alternatives are available. This was the case for the exemption granted in 2016 for the use of the SfMNPV virus to control *S. frugiperda*. The registration process for baculovirus-based biopesticides is streamlined in the US due to their low toxicity to non-target organisms and minimal environmental impact. Data requirements often focus on the safety of production processes, the stability of the viral formulation, and efficacy against the target pest. As of 2024, nearly 30 baculovirus-based products have been registered by the EPA, corresponding to around a dozen different species of baculovirus. Companies like Certis, AgBiTech, and Andermatt have the largest number of registered products in this region, targeting economically significant pests such as *H. armigera* and *S. frugiperda*, as previously mentioned. Unique offerings in this market include Lecontvirus WP from Andermatt Canada (formerly Sylvar Technologies) to control *Neodiprion abietis* in coniferous trees and the USDA Forest Service’s Gypchek WP for *Lymantria dispar*, evidencing a nuanced approach to pest management across diverse ecosystems. This growing number of registrations demonstrates the increasing acceptance and utility of baculoviruses in modern pest management practices.

### 4.2. Brazil

At least one third of the most sold pesticides in LATAM are classified as highly hazardous pesticides [224]. In this region, it is necessary to increase public health surveillance and improve monitoring systems for pesticide use, favoring the correct use of the products by small farmers [225,226]. In South America, each country adopts distinct strategies for biopesticide regulation. Among the countries that most promote the use of biopesticides is Brazil [227]. In fact, one of the most successful IPM programs in the world was implemented for soybean in Brazil during the 1970s [228]. In recent years, Brazil has revised its legislation to simplify and expedite the registration of biopesticides. This shift was partly driven by the arrival of *H. armigera* in the country, its resistance to classical pesticides, and the successful use of the HearNPV discussed previously, fostering the adoption of biopesticides as alternatives to chemical pesticides. As one of the largest pesticide consumers globally, Brazil has established distinct regulatory pathways for chemical and biological products, managed by the Ministry of Agriculture (MAPA), the Brazilian Health Regulatory Agency (Anvisa), and the Brazilian Institute for the Environment and Renewable Natural Resources (IBAMA). Notably, the registration process for pesticides used in organic agriculture is faster and less complex than for conventional agriculture, providing a significant advantage for biopesticides. Under Law 14,785/2023, two new ordinances have been introduced to streamline the registration process: Ordinance No. 02/2023 simplifies administrative procedures for modifying registrations, while Ordinance No. 03/2023 addresses pending applications, including “clone” products with identical characteristics or active ingredients. These advancements support the implementation of IPM programs and reflect Brazil’s commitment to sustainable agricultural practices.

By 2024, multiple baculovirus-based products were registered in Brazil, targeting pests like *S. frugiperda* and *H. armigera*. These products are being used in major agricultural sectors, including soybean, maize, and cotton cultivation. Brazil has observed a rapid growth in the biopesticide sector in the last few years. Companies operating in Brazil are increasingly adopting biocontrol methods, integrating baculovirus-based products into pest management strategies to combat resistance to chemical pesticides. This trend reflects the growing acceptance of environmentally friendly solutions in Brazilian agriculture.

### 4.3. European Union and UK

Until 31 December 2020, the EU and the UK shared a common regulatory framework for biopesticides under EU law. This shared regulatory structure ensured harmonized procedures for the evaluation, approval, and use of biopesticides across member states, including the UK. However, following the UK’s departure from the EU, their regulatory paths diverged, with the EU maintaining its framework under Regulation (EC) No 1107/2009, while the UK established its own independent rules. This shift marks a significant regulatory evolution, potentially influencing market access, innovation, and the adoption of biopesticides in each region.

#### European Union

The application of pesticides has been strictly controlled by community legislation since 1991 (Regulation (EC) No 1991/2002). Today, Regulation (EC) No. 1107/2009, issued by the European Parliament and the Council on 21 October 2009, governs the placement of plant protection products and the active substances contained therein. The aim of this regulation is to ensure a high level of protection of human and animal health and the environment while safeguarding the competitiveness of agriculture. Article 4 of the Regulation sets out the criteria for the approval of active substances by restricting the use and residues of plant protection products containing those active substances which may have harmful effects on humans, animals, and plants, the environment, or groundwater. It also addresses the environmental fate and distribution of the active substance, as well as its impact on non-target species, biodiversity, and the ecosystem. The latest data in the European Union’s pesticide database total 870 unauthorized active substances, compared with 466 authorized active substances.

As part of the Green Deal (COM/2019/640 final), the European Commission has proposed to reduce the use and risk of chemical pesticides by 50% by 2030 and to reduce the use of more hazardous pesticides by 50% by 2030. However, in the EU, the number of registered biopesticide active substances is fewer than in the USA, India, Brazil, or China because of the complexity of EU-based biopesticide regulations. In 2018, the volume of active substances sold across the Member States was notable in France, Spain, Germany, and Italy [229]. The EU promotes IPM, where sustainable biological, physical, and other non-chemical methods must be preferred if they provide satisfactory pest control. However, in 2019, chemical pesticides still accounted for 99.84% of pesticide sales compared to non-chemical biopesticides [230]. Despite this, the biopesticides market is anticipated to grow globally to USD 14.46 billion by 2028 with a compound annual growth rate (CAGR) of 18.2% [231]. To shorten the registration times for biopesticides and increase the availability of safer effective products, the second annex of Regulation (EC) Nº 1107/2009 was modified in 2022.

Furthermore, member states can authorize, in special circumstances, the commercialization of a pesticide for a maximum of 120 days. Emergency authorizations are regulated by Article 53 of Regulation (EU) 1107/2009 and allow for the use of products that are not (or not yet) authorized. Authorization may concern cases such as the use of an approved product on a crop not included in the label or the use of a non-approved product in cases where no (or insufficient) approved alternatives exist. This was the case for the use of the Se-MNPV in Almeria (Spain) before product approval.

As of 2024, only four baculovirus-based active substances have been registered within the European Union, with two additional approvals currently pending. This translates into a limited market presence, with just seven baculovirus-based biopesticide products available for agricultural use. Most of these products are manufactured by Andermatt Biocontrol. The first baculovirus to be registered in this region was the CpGV, which has been approved for use since 1 May 2009. Other approved baculoviruses include the HearNPV, SpliMNPV, and SeMNPV. This limited number highlights the regulatory and market challenges for expanding the use of such highly specific and environmentally friendly biopesticides in the EU.

### 4.4. China

Although China is the world’s largest pesticide user, the Chinese government has only promoted IPM programs since the late 1970s [232,233]. The Institute for the Control of Agrochemicals (ICAMA), under the Ministry of Agriculture and Rural Affairs, oversees national pesticide registration and compliance [234]. Recent regulatory updates include the release of revised administrative measures for pesticide registration, production licensing, labeling, and business licensing. China recently banned four highly toxic pesticides: omethoate, carbofuran, methomyl, and aldicarb (Announcement No. 736). The 10th National Pesticide Registration Review Committee was also established to ensure comprehensive and impartial reviews. In this region, all pesticides must be registered before they can be produced, sold, or used. In formulating regulations for biopesticides, there are distinct and more lenient policies compared to those governing chemical pesticides [233]. China also participates in international programs such as ADOPT-IPM to optimize and develop IPM tools [234].

The registration process for baculovirus-based biopesticides in China is complex, requiring extensive biological and toxicity testing conducted by qualified laboratories within the country. Despite these challenges, there are commercial products on the market that contain baculoviruses such as the HaNPV, SeMNPV, and SfMNPV as active ingredients. Additionally, some companies offer products combining baculoviruses with other microbial agents or synthetic insecticides, therefore expanding their host spectrum. Specialized consultancy firms assist foreign companies in navigating the regulatory requirements which can sometimes be intricate, with up-to-date information and regulations not always being easily accessible or clearly defined.

### 4.5. Global Biopesticide Regulation: A Comparative Summary

The global landscape for biopesticide regulation is characterized by significant diversity and complexity, reflecting the unique priorities and challenges faced by different regions. One of the key differentiators is the level of regulatory complexity and support for biopesticide registration.

The United States, Brazil, Europe, and China are the leading countries in the biopesticide market, each with distinct regulatory frameworks for the registration of biopesticides. In Brazil, the regulatory framework is notably more accommodating for biopesticides compared to other regions. The country has implemented distinct and more lenient registration processes for biopesticides, especially for organic agriculture, making the registration process faster and more cost-effective than in other regions. This approach contrasts sharply with the United States and the European Union, where the registration of biopesticides, although facilitated through specific provisions, remains rigorous and time-consuming due to extensive safety and environmental assessments. In Asia, China is adapting regulatory frameworks to balance rapid agricultural development with safety. However, China’s regulatory framework is complex, requiring detailed testing in local laboratories, which makes the process slower and more arduous.

In summary, while regions like Brazil lead in facilitating biopesticide registration through streamlined processes and supportive regulations, the United States and the European Union maintain more stringent frameworks to ensure comprehensive safety and efficacy evaluations. These factors make the Brazilian market particularly attractive for baculovirus-based products, as its relatively faster and less expensive registration process, combined with the growing demand for sustainable pest control solutions, presents significant opportunities for the industry. These comparative insights underscore the global effort towards sustainable pest management, with varying degrees of regulatory complexity and support shaping the adoption and promotion of biopesticides.

## 5. Conclusions

This review establishes that baculovirus-based biopesticides present a scientifically sound and environmentally sustainable option for managing economically significant lepidopteran pests. Their unique infection mechanism—through the ingestion of occlusion bodies—ensures exceptional target specificity with minimal impact on non-target organisms. However, the relatively slow speed of kill in comparison to chemical insecticides remains a key limitation, underscoring the need for improved strain selection and genetic refinements. Furthermore, while baculoviruses show excellent efficacy under controlled conditions, their field performance can sometimes fall short of immediate pest suppression requirements in high-pressure agricultural systems. This discrepancy is mainly due to their intrinsic infection kinetics, which generally require 4–7 days to cause mortality. Nonetheless, this limitation can often be offset through timely application, integration with other strategies (e.g., Bt products or selective chemicals), and formulation improvements that enhance persistence and uptake. These technological and strategic developments continue to narrow the gap between laboratory efficacy and field performance, thereby supporting their growing adoption in integrated pest management programs.

The current production methods, particularly cost-effective in vivo systems, are hampered by challenges such as yield variability and contamination risks. Standardizing these production protocols and applying advanced genetic characterization techniques are essential steps to secure consistent product quality and optimize field performance. Despite recent advances in formulation technologies—such as microencapsulation and the incorporation of UV protectants and stabilizers—which have improved field persistence and efficacy, none of the formulations available to date provide adequate stability under ambient storage conditions. This limitation in shelf life remains a critical obstacle for broader commercial distribution.

An analysis of the target pest spectrum reveals that extensive research and development efforts have generated a wide range of baculovirus formulations for high-impact pests such as *H. armigera*, *S. frugiperda*, and *C. pomonella*. In contrast, considerably fewer baculovirus products are registered for pests like *M. brassicae*, *O. nubilalis*, *S. littoralis*, and *P. xylostella*. This imbalance likely reflects a combination of factors, including regional differences in pest pressure, economic return on investment, and technical challenges related to host–virus specificity and product development. In some cases, the lower commercial interest may stem from the availability of other effective pest management strategies, but the lack of baculovirus-based options nonetheless highlights a gap in biocontrol diversification for these species.

Regional differences are also critical to understanding the global landscape. South America, facing intense pest pressures and benefiting from more flexible regulatory environments, supports a broad and growing portfolio of baculovirus products. Conversely, in Europe, stringent regulatory requirements have limited approvals predominantly to granulovirus formulations targeting *C. pomonella*, thereby constraining product diversity. In regions such as Asia and North America, market trends are characterized by a mix of target-specific and integrated formulations, influenced by local pest challenges and regulatory practices.

Although baculoviruses have shown considerable potential in integrated pest management, several key knowledge gaps remain. First, the molecular mechanisms governing host–pathogen specificity are still not fully understood, limiting the ability to expand host range or enhance virulence. Second, there is a lack of diversity in formulation types, which restricts their adaptability to different field conditions. Third, in vitro production methods are often cost-prohibitive and technically challenging, impeding large-scale manufacturing. Finally, the absence of standardized international quality control guidelines hampers the broader commercialization and regulatory harmonization of baculovirus-based products. Future research should address these limitations by developing genetically enhanced viral strains, optimizing delivery systems, reducing production costs, and establishing robust quality assurance protocols.

Divergent regulatory frameworks play a pivotal role in determining market dynamics. While European standards, which demand extensive safety and efficacy data, restrict the diversity of registered products, more accommodating regulatory systems in South America and parts of Asia have facilitated wider product registration. This regulatory variability not only affects product availability but also shapes future research and development priorities.

Despite the advances reviewed here, a gap still exists between research development and practical implementation. This is particularly evident when comparing laboratory performance with field efficacy, the production scalability of existing virus preparations, and their alignment with on-the-ground application requirements. Addressing this disconnect will require targeted efforts to reduce costs, improve quality control systems, and ensure that product formulations and delivery methods meet the specific needs of farmers in diverse agricultural contexts.

## Figures and Tables

**Table 1:** Registered baculovirus products targeting *C. pomonella*, associated host crops, and recommended dose.

Products Available in Market	Registration/Sale/Distribution Companies	Dose/ha
CAPEX	Andermatt Biocontrol	5 × 10^12^ OBs/ha
GRANUPOM	Biobest	-
CARPOVIRUSINE	UPL	10^13^ OBs/ha
CARPOVIRUSINE EVO2		
CYD X LC	Certis	3 × 10^12^–1.2 × 10^13^ OBs/ha
CYD-X		6.6 × 10^12^–9.9 × 10^12^ OB/ha
CYD-X XTRA		3 × 10^12^ OBs/ha
MADEX	Andermatt/Agri Check S.R.L./Key Industries Ltd.	3 × 10^12^ OBs/ha
MADEX TWIN		
VIREX	Grochem	3 × 10^12^ OBs/ha
EN VIVO SC	Point Andina	1.4 × 10^11^–9 × 10^12^ OBs/ha

**Table 2:** Registered baculovirus products targeting *H. armigera*, associated host crops, and recommended dose.

Products Available in Market	Registration/Sale/Distribution Companies	Dose/ha
HELICOVEX	Andermatt	1.5 × 10^12^ OBs/ha
VERPAVEX		
HELIGEN	AgBiTech	3.8 × 10^11^–1.3 × 10^12^ OBs/ha
SURTIVO PLUS		1.4 × 10^11^–1.7 × 10^12^ OBs/ha
SURTIVO SOJA		
VIVUS ARMIGEN		3.7 × 10^11^–9.7 × 10^11^ OBs/ha
DIPLOMATA EVO	Koppert	3.7 × 10^11^–1.5 × 10^12^ OBs/ha
HELI-CIDE	Pest Control India	-
GEMSTAR LC	Certis/Mitsui & Co	1.5 × 10^12^ OBs/ha
GEMSTAR MAX	Mitsui & Co	-
HELIOKILL	Ajay Biotech	-
VPN ULTRA	Agricola el Sol	-
HELITEC	Elephant vert	2.5 × 10^12^ OBs/ha
HzNPV CCAB	CCAB Agro S.A.	7.5 × 10^11^–1.5 × 10^12^ OBs/ha
SpliNPV + 3% Betacypermethrin	Unioasis	1.1 × 10^10^–1.5 × 10^10^ OBs/ha

**Table 3:** Registered baculovirus products targeting *P. xylostella*, associated host crops, and recommended dose.

Products Available in Market	Registration/Sale/Distribution Companies	Product Dose/ha
EN VIVO SC	Point Andina	1.4 × 10^11^–9 × 10^12^ OBs/ha
PLUTEX	Andermatt	2.5 × 10^12^ OBs/ha
BYPEL 1	Unioasis	-
SeNPV + Bt		-
VPN ULTRA	Agricola el Sol	-
LEPIGEN	Agbitech	1.3 × 10^12^ OBs/ha

**Table 4:** Registered baculovirus products targeting *S. frugiperda*, associated host crops, and recommended dose.

Products Available in Market	Registration/Sale/Distribution Companies	Dose/ha
CARTUCHOVIT	Vitae Rural Biotecnologia	3 × 10^12^ OBs/ha
SURTIVO PLUS	Agbitech	1.4 × 10^11^–1.7 × 10^12^ OBs/ha
SURTIVO ULTRA		
FAWLIGEN		3.7 × 10^11^–2.2 × 10^12^ OBs/ha
CARTUGEN		3.7 × 10^11^–1.5 × 10^12^ OBs/ha
EN VIVO SC	Point Andina	1.4 × 10^11^–9 × 10^12^ OBs/ha
SPODOVIR PLUS	Andermatt	5 × 10^10^–1 × 10^11^ OBs/ha
VIRCONTROL-SF	Simbiose Agro Tecnología Biológica	4.3 × 10^11^ OBs/ha
VPN ULTRA	Agricola el Sol	-
